# Ultrastructural changes in cristae of lymphoblasts in acute lymphoblastic leukemia parallel alterations in biogenesis markers

**DOI:** 10.1186/s42649-021-00069-4

**Published:** 2021-12-29

**Authors:** Ritika Singh, Ayushi Jain, Jayanth Kumar Palanichamy, T. C. Nag, Sameer Bakhshi, Archna Singh

**Affiliations:** 1 Department of Biochemistry, Teaching Block, All India Institute of Medical Sciences, Room 3044, New Delhi, 110029 India; 2 Department of Biochemistry, Convergence Block, All India Institute of Medical Sciences, New Delhi, India; 3 Department of Anatomy, Teaching Block, All India Institute of Medical Sciences, New Delhi, India; 4 Department of Medical Oncology, BRAIRCH, All India Institute of Medical Sciences, New Delhi, India

**Keywords:** Acute lymphoblastic leukemia, Electron microscopy, Mitochondrial morphology, Cristae, Mitochondrial biogenesis

## Abstract

We explored the link between mitochondrial biogenesis and mitochondrial morphology using transmission electron microscopy (TEM) in lymphoblasts of pediatric acute lymphoblastic leukemia (ALL) patients and compared these characteristics between tumors and control samples. Gene expression of mitochondrial biogenesis markers was analysed in 23 ALL patients and 18 controls and TEM for morphology analysis was done in 15 ALL patients and 9 healthy controls. The area occupied by mitochondria per cell and the cristae cross-sectional area was observed to be significantly higher in patients than in controls (*p*-value = 0.0468 and *p*-value< 0.0001, respectively). The mtDNA copy numbers, *TFAM*, *POLG,* and *c-myc* gene expression were significantly higher in ALL patients than controls (all *p*-values< 0.01). Gene Expression of *PGC-1α* was higher in tumor samples. The analysis of the correlation between *PGC-1α* expression and morphology parameters i.e., both M/C ratio and cristae cross-sectional area revealed a positive trend (*r* = 0.3, *p* = 0.1). The increased area occupied by mitochondria and increased cristae area support the occurrence of cristae remodelling in ALL. These changes might reflect alterations in cristae dynamics to support the metabolic state of the cells by forming a more condensed network. Ultrastructural imaging can be useful for affirming changes occurring at a subcellular organellar level.

**Supplementary Information:**

The online version contains supplementary material available at 10.1186/s42649-021-00069-4.

## Introduction

Acute Lymphoblastic Leukemia (ALL) resulting from malignant transformation and proliferation of lymphoblasts (lymphoid progenitor cells) in the bone marrow, blood, and extramedullary sites constitutes sixty to eighty-five percent of all childhood leukemias reported globally and in India (Arora and Arora 2016; Terwilliger and Abdul-Hay 2017; Cárceles-Álvarez et al. 2017).

Mitochondria, the alphaproteobacterial endosymbiont, constitute one of the indispensable intracellular organelles in tissues, due to the plethora of diverse functions regulated by them inside the cell (Shokolenko and Alexeyev 2015; Pánek et al. 2020; Bock and Tait 2020; Lee et al. 2019). Mitochondria have become an important focus of cancer research as altered metabolic reprogramming has been identified as a hallmark of cancer (Hanahan and Weinberg 2000; Hsu et al. 2016; Jing et al. 2019). A recent paper by Panuzzo et al., provides insights highlighting the role of mitochondria and the consequences of abnormal functioning of mitochondria in the generation of leukemic stem cells (Panuzzo et al. 2020; Roger et al. 2017).

Each human cell has hundreds and thousands of mitochondria with each carrying a variable number of mitochondrial DNA (mtDNA) copies (Ju et al. 2014; Wallace 2012). Findings from our lab have previously demonstrated a higher mtDNA copy number in lymphoblasts of pediatric ALL patients as compared to mononuclear cells of healthy controls (Jain et al. 2018). MtDNA replication and the transcription and translation of mtDNA encoded genes form an integral part of mitochondrial biogenesis. Peroxisome proliferator-activated receptor-gamma coactivator (*PGC-1α*) is the master transcriptional regulator of mitochondrial biogenesis and interacts with both nuclear respiratory factors (*NRF1 and NRF2*), that have a role in the replication and transcription of mtDNA and enzymes of energy metabolism. Proper mitochondrial function is a result of a tightly coordinated balance between its biogenesis and degradation (mitophagy) (Zhang and Xu 2016; Ploumi et al. 2017).

The relationship between mitochondrial morphology, its dynamics, its role in mitochondrial homeostasis, and implications of disruption of this homeostasis in cancer has been investigated (Alirol and Martinou 2006; John et al. 2005; Chen et al. 2005). The inner membrane of mitochondria is formed from two structures: inner boundary membrane (IBM) and cristae and cristae junctions (CJs) form the connecting link between the two. Optic atrophy gene 1 (OPA1), the mitochondrial contact site and cristae organizing system (MICOS), and ATP synthase have been identified as the main regulators of cristae dynamics (Kondadi et al. 2019; Kondadi n.d.; Baker et al. 2019). Mitochondrial cristae are known to transition between a ‘condensed state’, with a dense cristae network, and an ‘orthodox state’ consisting of expanded cristae morphology (Cogliati et al. 2016). Such cristae remodeling is an effective way for mitochondria to optimize performance or response to stimuli such as oxidative stress or apoptosis (Mannella 2008). Cristae possess oxidative phosphorylation (OXPHOS) complexes and super-complexes, which function as respiratory bioreactors. Changes in cristae dynamics can affect the performance of the electron transport chain (ETC), affect supercomplexes formation and function. These changes in cristae dynamics have been linked with pathological conditions including cancer (Cogliati et al. 2016; Vyas et al. 2016; Chan 2020; Simula et al. 2017; Chen and Chan 2017).

Modulation of mitochondrial mass and mtDNA copy numbers have been observed in leukemias including ALL but the status of cristae remodeling as observed by electron microscopy (EM) technique and linkages with mitochondrial biogenesis in ALL has not been documented in much detail. Our study attempts to highlight alterations observed at the ultrastructural level in mitochondria of the lymphoblasts of pediatric ALL samples in the context of higher mtDNA copy numbers. We also present a way to combine imaging and molecular biology for a better, composite understanding of links between mitochondrial form and function in leukemia biology.

## Methodology

### Patients and controls

A total of 23 newly diagnosed, untreated ALL patients recruited from the pediatric oncology Outpatient Department at Dr. B.R.A.I.R.C.H, AIIMS, New Delhi between March and December 2019, were included in the study. The study was approved by the Institutional Ethical Committee: Ref. No. IECPG-112/28.02.2019. Control samples included peripheral blood (PB) of 5 pediatric healthy siblings of ALL patients and bone marrow (BM) samples of another 13 pediatric patients with solid malignancy, who underwent bone marrow aspiration as part of staging workup with no subsequent detection of marrow involvement by primary malignancy. Informed consent from all participants or their guardians was taken before their recruitment into the study. The confidentiality was maintained throughout the study using appropriate measures.

### Sample collection and processing

The diagnosis of all patients was confirmed to be ALL from the flow cytometric and morphological reports of the BM samples. Samples for experimental protocols were obtained during the routine draw being done for diagnosis after sample aliquots had been allocated for diagnostic workup. All samples were collected in vacutainers containing the anticoagulant, EDTA. Mononuclear cells were obtained by whole blood lysis using RBC lysis buffer in a ratio of 1:3 respectively. The mononuclear pellet was divided into multiple aliquots for the different experiments, detailed below.

### Mitochondrial DNA copy number estimation

The total cellular DNA extraction from mononuclear cells was performed using a protocol for isolation of nuclear and mtDNA as previously mentioned (Fukuoh et al. 2014). The DNA was quantified using a Qubit fluorometer. The quality was assessed by the A260/280 ratio and on agarose gel electrophoresis. Relative mtDNA copy number estimation was done by fluorescence-based quantitative real-time PCR (qPCR) using DNA-binding dye SYBR green in Bio-Rad CFX96 Real-Time PCR machine system for all samples. The mitochondrial gene copy number was normalized using nuclear gene beta-actin. Gene-specific primer sequences used for the qPCR for copy number estimation are listed in Table S1. The mtDNA copy number, normalized to nuclear copies per cell, in each subject and control sample was calculated using the following formula:

$$\mathbf{2}\hat{\mkern6mu} \left[\mathbf{Ct}\ \left(\boldsymbol{\upbeta} -\mathbf{actin}\right)-\mathbf{Ct}\ \left(\mathbf{minor}\ \mathbf{arc}\right)\right]$$

### Transmission electron microscopy

#### Sample processing

The mononuclear cell sample from BM/ PB was fixed in 2.5% glutaraldehyde and 2% paraformaldehyde in 0.1 M sodium phosphate buffer (pH 7.4) for 4–6 h at 4 °C. The samples were postfixed in 1% OsO_4_, dehydrated in acetone, infiltrated, and embedded in Araldite CY 212 (TAAB, UK). Thick sections (1 μm) were cut with an ultramicrotome (Leica Ultracut UC7, Austria), mounted on to glass slides, stained with aqueous toluidine blue, and observed under a light microscope for gross observation of the cells/tissue and quality of tissue fixation. For EM examination, thin sections of grey-silver color interference (70–80 nm) were cut and mounted onto 300 mesh- copper grids. Sections were stained with aqueous uranyl acetate and alkaline lead citrate and observed under a Tecnai G2 20 high-resolution transmission electron microscope (Fei Company, The Netherlands) at an operating voltage of 200 kV. Images were digitally acquired using Digital Micrograph (Gatan, Inc.) software attached to the microscope. The processing of the samples and imaging was done at the Sophisticated Analytical Instrumentation Facility, AIIMS, New Delhi.

#### TEM image analysis for mitochondrial morphology

Out of the 23 patients’ samples and 18 control samples obtained, 15 and 9 samples respectively, were found to be of suitable quality/ quantity for TEM analysis after preliminary processing. Lymphoblasts and lymphocytes were identified based on a high nuclear to cytoplasmic ratio, i.e., N/C ratio ≥ 1. The quality check for the obtained images was performed by an in-house EM specialist. All the images were taken at low magnification (2250X) and the same images were also captured at higher magnification (4000X and 9900X). All images were selected and approved for further analysis by the TEM expert.

**Fig. 1:**
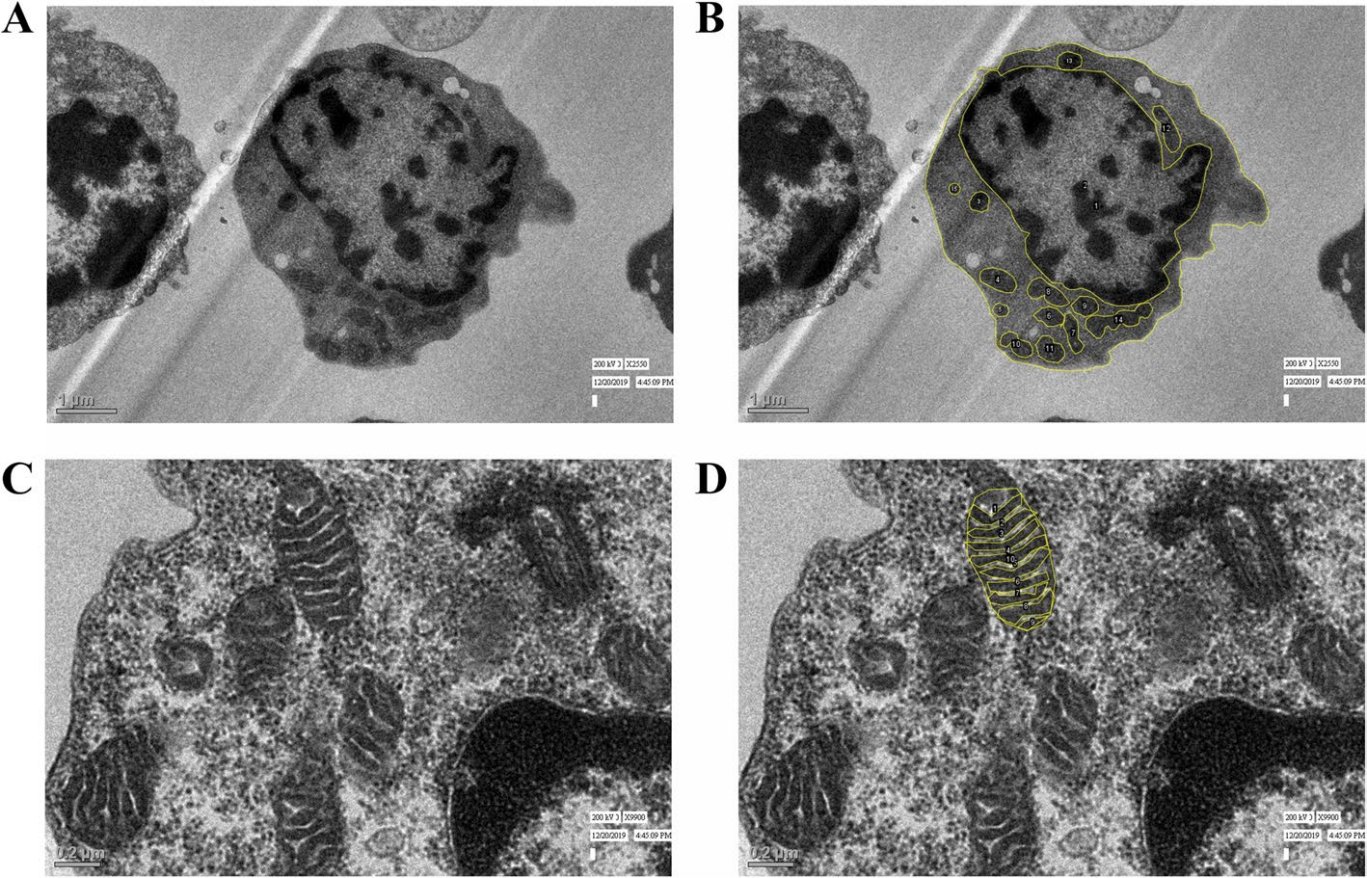
Transmission electron microscopy images of lymphoblasts in bone marrow samples of ALL patients. The image analysis was done using ImageJ software after calibrating the images by the use of scales on the figure. **A** Representative images of electron microscopy of lymphoblast of a B-ALL patient (2250X; scale bar = 1 μm). **B** The mitochondria have been traced using ImageJ tool for calculation of area occupied by mitochondria in the same section mentioned in **A**. **C** Representative images of electron microscopy of mitochondria in lymphoblast of a B-ALL patient at higher magnification (9900X; scale bar = 0.2 μm). **D** The cristae of the mitochondria have been traced using the ImageJ tool for calculation of cristae cross-sectional area in the same section mentioned in **C**

Image analysis was done using the freely available ImageJ software (NIH, USA). The image was first calibrated according to the scale mentioned on every image. The number of images captured in each sample ranged from 10 to 30, depending upon the quality of images taken. The area occupied by mitochondria was calculated in 5 representative sections in each patient and control by tracing the cell membrane, followed by tracing the nuclear membrane and individual mitochondrial membranes. The mitochondria/cytoplasmic area (M/C ratio) was calculated for each section and the mean M/C ratios were calculated for each patient. For cristae cross-sectional area, all suitable quality mitochondria in ≥7 fields of lymphoblasts in 15 ALL patients and lymphocytes in 9 controls taken at higher magnifications were analyzed. The mitochondria that appeared abnormally long or swollen were not included in the analysis. The cristae cross-sectional area was calculated by tracing each crista and subsequently tracing the outer mitochondrial membrane (OMM) and obtaining their ratio. This evaluation was done by two researchers individually on the TEM sections and corroborated by an EM specialist (Fig. 1).

### mRNA gene expression estimation

Total RNA was isolated from mononuclear cell pellets of ALL patients and controls using the trizol-chloroform method. Total RNA isolated was reverse transcribed to complementary DNA (cDNA) in an Eppendorf Mastercycler Personal thermal cycler using the following protocol: 25 °C for 10 min, 42 °C for 60 min, 70 °C for 10 min, and final hold at 4 °C. The resultant cDNA was diluted to 100 μl using nuclease-free water and used for further downstream experiments. Expression of the genes related to mitochondrial biogenesis, i.e., *TFAM* (mitochondrial transcription factor A), *POLG* (polymerase γ), and *PGC-1α* was assessed by qPCR. An estimation of mitochondrial function was done using *CS* (citrate synthase) gene expression (Cai et al. 2017). *c-myc* gene expression was also analyzed as it is a transcription factor with a role in mitochondrial biogenesis (Li et al. 2005). Primer sequences for gene expression analysis have been listed in Table S2. A fluorescence-based quantitative real-time PCR using DNA-binding dye SYBR green method was used for gene expression analysis Agilent AriaMx Real-Time PCR machine system using PCR cycling conditions listed here: 5 min at 95 °C, 30 s at 95 °C, 62 °C for 35 cycles, 30 s at 72 °C, 5 min at 72 °C. The mRNA gene expression was normalized using beta-actin as the housekeeping gene.

### Statistical analysis

General patient demographic variables were summarized using descriptive statistics and appropriate measures of central tendency e.g., mean or median. Nonparametric statistical tests were applied using GraphPad Prism 6 software (San Diego, CA, USA). Mann-Whitney U test was used to compare mtDNA copy number, M/C ratios, cristae cross-sectional area, and mRNA gene expression analysis between patients and controls (unpaired samples). Spearman correlation analysis was used for the comparison of biogenesis markers with corresponding M/C ratios and cristae cross-sectional area. A *p*-value of < 0.05 was considered to be statistically significant.

## Results

### Patient characteristics

The patients were coded ALL 1 to ALL 23 for recording the demographic details which have been listed in Table 1. The BM controls had a mean age of 7.9 years (range 1–17 years), with 8 males and 5 females in the study control group. The siblings had a mean age of 12.6 years (range 7–18 years) with 2 females and 3 males in the study group.

**Table 1:** Descriptive table listing the demographic details of the ALL patients

Descriptive variables	***n*** = 23
**Sex**	
Female	*n* = 5
Male	*n* = 18
**Age (years)**	
Mean	9.4
Range	2–18
**Subtype**	
B-ALL	*n* = 14
T-ALL	*n* = 9
**WBC (cells/ul)**	
Mean	179,468.3
Range	9230–1,150,000
**Hb (g/dl)**	
Mean	7.08
Range	1.7–14.5
**Chromosomal translocation**^**a**^ Absent	*n* = 9
BCR-ABL 1	*n* = 2
ETV6-RUNX1	*n* = 3
E2A-PBX1	*n* = 2
MLL-AF4	*n* = 1

*WBC* Whole white blood cell count, *Hb* Haemoglobin, *BM* Bone marrow

^a^The report for chromosomal translocation for 6 patients were not available

### Features of mitochondria in lymphoblasts of ALL patients

**Fig. 2:**
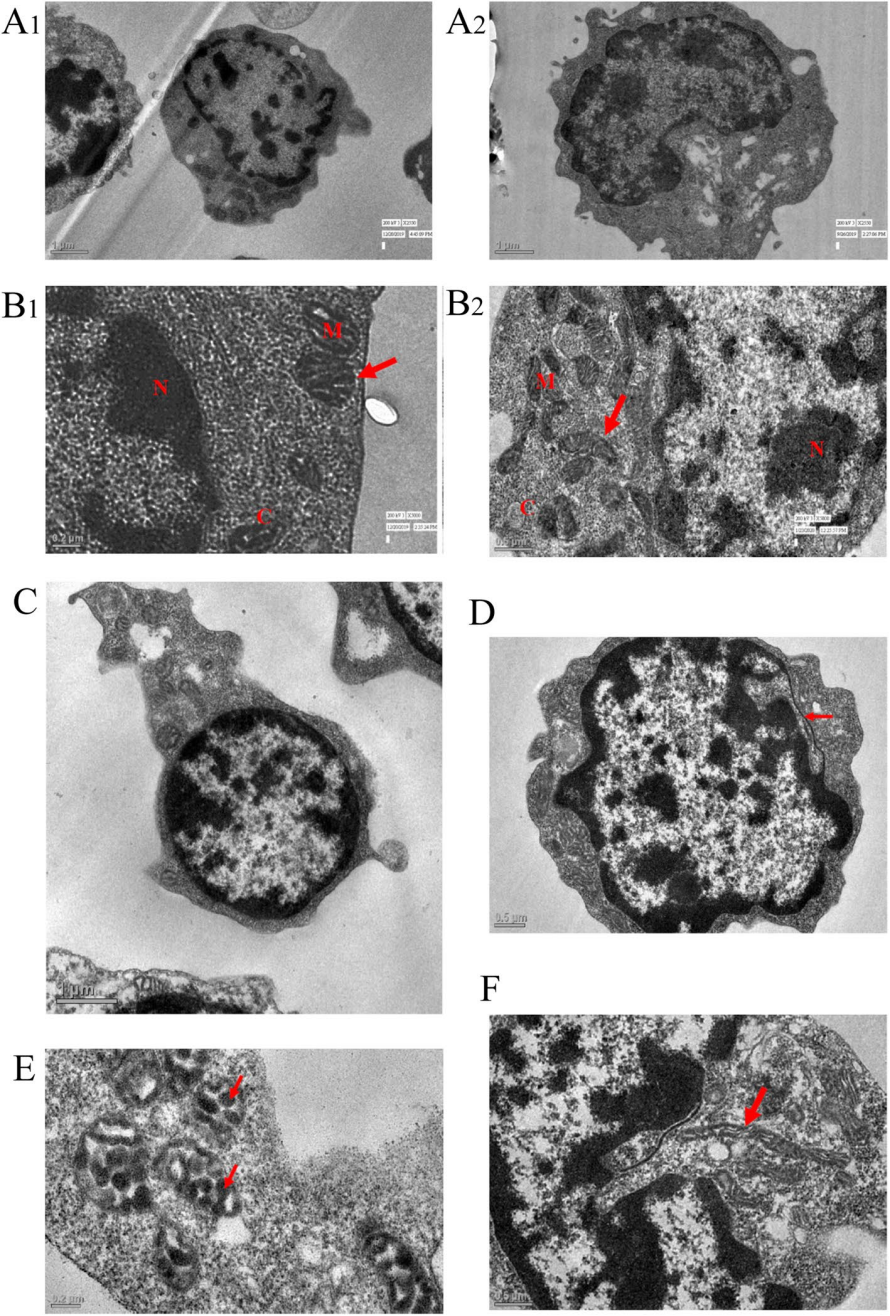
**A** Transmission electron microscopy images of mitochondria in lymphoblast of a B-ALL patients and lymphocytes of healthy controls in **A1** and **A2** respectively (2250X; scale bar = 1 μm). **B.1** Representative electron microscopy image of mitochondria in lymphoblast of a T-ALL patient (5000X; scale bar = 0.2 μm). The mitochondria are round and globular. **B.2** Representative electron microscopy image of variously shaped mitochondria in lymphoblast of a B-ALL patient (5000X; scale bar = 0.5 μm). **C** Representative electron microscopy image of hand-mirror shaped lymphoblast in a B-ALL patient (2250X; scale bar = 1 μm). **D** Representative electron microscopy image of lymphoblast showing nuclear pockets in a B-ALL patient (4000X; scale bar = 0.5 μm). **E** Representative electron microscopy image of lymphoblast showing vesicular cristae in mitochondria of a T-ALL patient; each arrow depicting cristae in mitochondria (9900X; scale bar = 2 μm). **F** Representative electron microscopy image of lymphoblast in which the arrow is showing abnormally elongated mitochondria in a B-ALL patient (4000X; scale bar = 0.5 μm). N = nucleus; C = cytoplasm and M = mitochondria

TEM was performed on 24 specimens: 15 ALL patients and 9 healthy controls. Images of lymphoblast in ALL patients and lymphocytes in controls were captured for analysis (Fig. 2:A1 and A2). Mitochondria were divided into two types: a) regular mitochondria with tubular or lamellar cristae, observed in > 95% sections of lymphoblast of ALL patients (Fig. 2:B1 and B2) and b) vesicular mitochondria in which the inner membrane encloses a vesicular matrix and cristae are enlarged, observed in lymphoblast sections of one ALL patient (Fig. 2E). Hand mirror-shaped lymphoblasts were seen in one patient and nuclear pockets were observed in three patients (Fig. 2C and D respectively). These features of mitochondria have been previously reported in ALL (Sharp et al. 1979; Stekhoven 1986). Abnormally elongated mitochondria were also observed in more than five sections in patients’ lymphoblasts (Fig. 2F). B and T subtype ALL patients showed no apparent differences in the mitochondrial and cristae morphology.

### Area occupied by mitochondria per cell and cristae cross sectional area

**Fig. 3:**
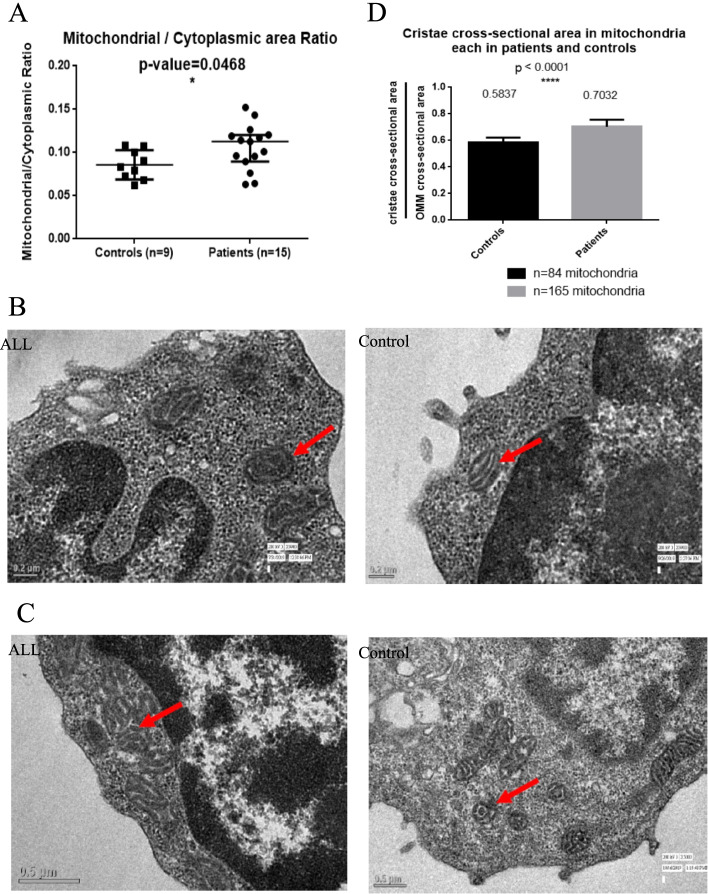
**A** Scatter plot representation of average M/C ratio in ALL patients (*n* = 15) and controls (*n* = 9); *p* = 0.046. Transmission electron images were analyzed for qualitative differences between ALL patients and healthy controls; arrow depicting mitochondria in the TEM images. **B** Thicker and denser cristae in mitochondria in lymphoblast of a T-ALL patient (left; 9900X; scale bar = 0.2 μm) as compared to lymphocyte of BM control (right; 9900X; scale bar = 0.2 μm). **C** Thicker and denser cristae in mitochondria in lymphoblast of a B-ALL patient (left; 5000X; scale bar = 0.5 μm) as compared to lymphocyte of patient’s sibling (right; 5000X; scale bar = 0.5 μm). **D** Bar graph representation of cristae cross-sectional area in mitochondria (*n* = 165) of lymphoblasts of 15 ALL patients versus mitochondria (*n* = 84) in lymphocytes of 9 healthy control along with medians depicted on top of the bars in each group; *p* < 0.0001. These numbers 165 and 84 suggests multiple mitochondria in each section were analysed in ALL patients and controls (* *p* < 0.05, ** *p* < 0.01, *** *p* < 0.001, Mann-Whitney U test). OMM- Outer Mitochondrial Membrane

Table 2 summarizes the average N/C and M/C ratios obtained by TEM evaluation of the lymphoblast sections from the bone marrow of ALL patients and lymphocytes of controls, respectively. The M/C ratio was significantly higher in ALL patients as compared to controls (*p* = 0.0468; median: 0.1127 and 0.0832 of ALL patients and controls respectively; Fig. 3A). Cristae appeared compact and denser in mitochondria from lymphoblasts of ALL patients as compared to mitochondria in lymphocytes of BM controls and patients’ sibling controls as seen in TEM analysis (Fig. 3B and C). All fields at higher magnifications had ≥1 mitochondrion. Cristae cross-sectional area was calculated in a total of 84 mitochondria in lymphocytes of 9 controls and 165 mitochondria in lymphoblasts of 15 ALL patients. The cross-sectional area was significantly higher in ALL patients as compared to controls in concordance with the visual analysis (median: 0.7032 and 0.5837 in ALL patients and controls respectively; *p* < 0.0001; Fig. 3D).

**Table 2:** Descriptive table showing the average N/C and M/C ratios recorded in lymphoblasts from sections of ALL patients and lymphocytes in controls

S.No.	Average N/C ratio	Average M/C ratio
ALL 1	1.162	0.120
ALL2	0.488	0.119
ALL 3	1.238	0.096
ALL4	1.493	0.090
ALL5	1.272	0.143
ALL6	1.250	0.101
ALL7	1.510	0.117
ALL8	1.190	0.096
ALL9	1.248	0.152
ALL10	1.209	0.063
ALL11	0.706	0.126
ALL12	0.970	0.113
ALL13	1.740	0.076
ALL14	0.763	0.114
ALL15	1.440	0.064
HBM1	0.898	0.073
HBM2	1.554	0.108
HBM3	0.810	0.068
HBM4	0.784	0.079
HBM5	0.762	0.062
SIB1	1.150	0.083
SIB2	0.810	0.108
SIB3	1.601	0.100
SIB4	0.919	0.091

*N/C* Nuclear cross-sectional area / cytoplasmic area and *M/C* Mitochondrial area/ cytoplasmic area, *HBM* Healthy Bone Marrow controls, *SIB* Patients’ siblings

### MtDNA copy number and gene expression in ALL patients and controls

**Fig. 4:**
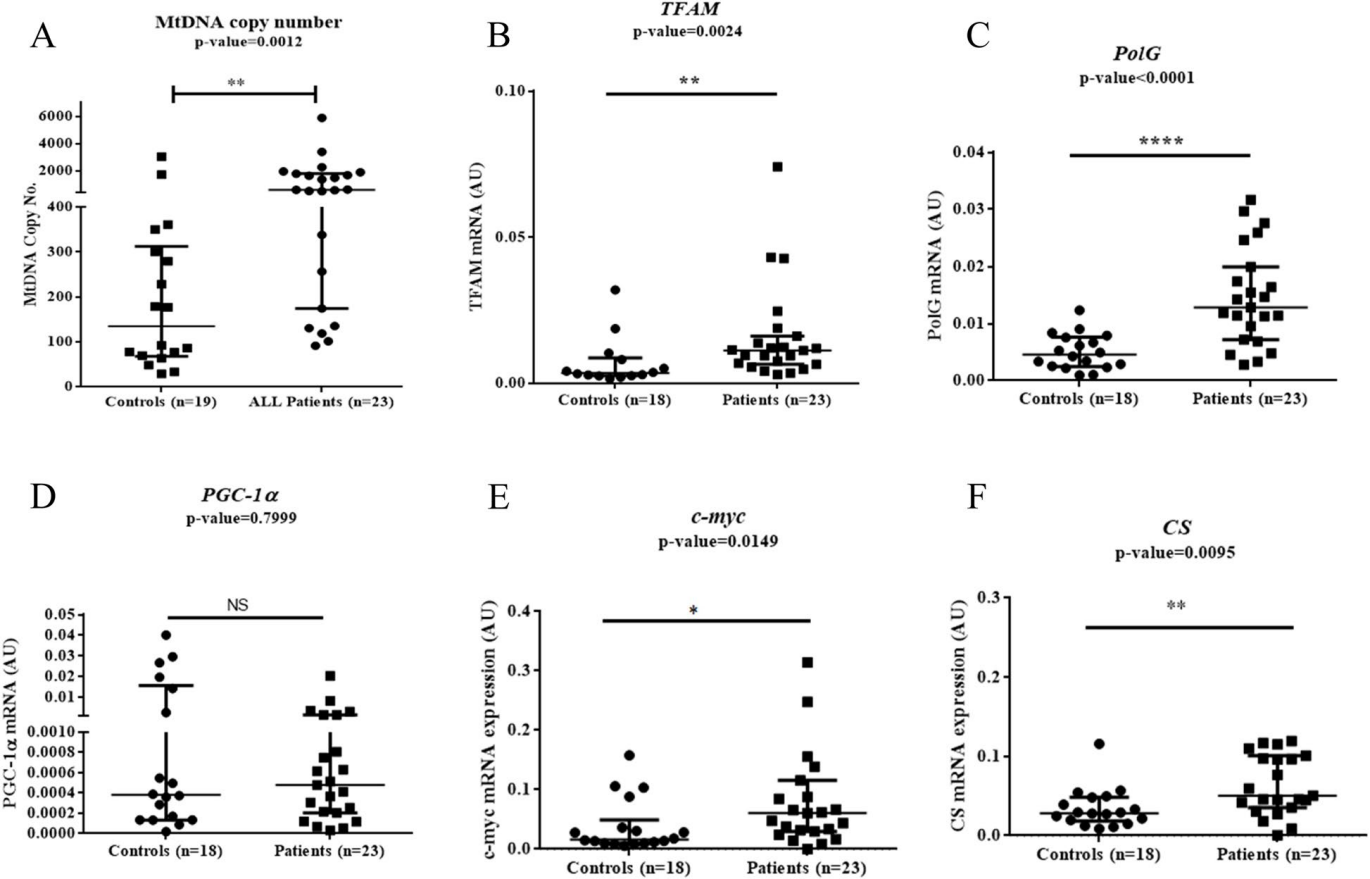
Scatter plots for mtDNA copy number and mRNA expression of *TFAM, POLG, PGC-1α*, *c-myc,* and *CS* genes in ALL patients and healthy controls. **A** MtDNA copy number analysis in ALL patients and healthy controls (*p* = 0.0012). **B** *TFAM* gene expression in ALL patients and healthy controls (*p* = 0.0024). **C** *POLG* gene expression in ALL patients and healthy controls *p* < 0.0001). **D** *PGC-1α* gene expression in ALL patients and healthy controls (*p* = 0.7999). **E** *c-myc* gene expression in ALL patients and healthy controls (*p* = 0.0149). **F** *CS* gene expression in ALL patients and healthy controls (*p* = 0.0095); (* *p* < 0.05, ** *p* < 0.01, *** *p* < 0.001, Mann-Whitney U test)

The mtDNA copy number was significantly higher in ALL patients as compared to healthy controls (median: 580.0 and 134.1 in ALL patients and controls respectively; *p* = 0.0012; Fig. 4A). The gene expression levels of *TFAM, POLG* and *c-myc,* and *CS* were significantly higher in ALL patients as compared to controls. Medians for *TFAM* gene expression were 0.011 AU and 0.003 AU in ALL patients and controls respectively (*p* = 0.0024; Fig. 4B). Medians for *POLG* gene expression were 0.013 AU and 0.004 AU in ALL patients and controls respectively (*p* < 0.0001; Fig. 4C). Medians for *c-myc* gene expression were 0.060 AU and 0.016 AU in ALL patients and controls respectively (*p* = 0.0149; Fig. 4E). Medians for *CS* gene expression were 0.050 AU and 0.028 AU in ALL patients and controls respectively (*p* = 0.0095; Fig. 4F). The median *PGC-1α* expression was higher in ALL patients than controls; however, the difference was not significant (median: 0.00048 AU and 0.00038 AU in ALL patients and controls respectively; *p* = 0.799; Fig. 4D). The expression of *TFAM* showed a strong positive correlation with *PGC-1α* expression (data not shown).

### Correlation between average M/C ratios, cristae cross-sectional area and biogenesis markers in ALL patients

We assessed the correlation between average M/C ratios, cristae cross-sectional area and biogenesis markers i.e., *TFAM, POLG, PGC-1α, c-myc*, *CS* in the 15 ALL patients in which the TEM analysis had been done (Table 3). The analysis of correlation of *PGC-1α* expression showed a positive trend with both M/C ratio and cristae cross-sectional area with *p*-values approaching significance. The trend was in the opposite direction for *c-myc*.

**Table 3:** Spearman correlation between biogenesis markers and cristae parameter in 15 ALL patients

	Average M/C ratio		Cristae cross sectional area	
	*r* value	*p* value	*r* value	*p* value
*POLG* gene expression	0.0	> 0.99	−0.32	0.25
*PGC-1α* gene expression	**0.36**	**0.18**	**0.36**	**0.19**
*TFAM* gene expression	−0.05	0.86	− 0.34	0.24
*c-myc* gene expression	**−0.36**	**0.18**	**−0.41**	**0.15**
*CS* gene expression	−0.28	0.32	**−0.42**	**0.13**

The bold typeface indicates *p*-values approaching significance

*POLG* Polymerase γ, *TFAM* Mitochondrial transcription factor A, *CS* Citrate synthase, *r* Spearman correlation coefficient

*p* < 0.05, ** *p* < 0.01, *** *p* < 0.001

## Discussion

While the Warburg hypothesis is still valid and tumors can undergo enhanced aerobic glycolysis, they also continue to maintain, and maybe upregulate, mitochondrial bioenergetic capacities (Tran et al. 2016; Zheng 2012; Koppenol et al. 2011). Irrespective of the exact phenotype of the tumor, metabolic dysfunction is a principal component of cancer with mitochondria as the main metabolite hub inside the cell (Frezza 2017; Gammage and Frezza 2019). This metabolic dysfunction affects the cellular bioenergetics, leading to pro-tumoral metabolic transformation and promotion of cancer proliferation and triggers tumor-promoting (epi)-genetic changes mediated by various metabolites (Gammage and Frezza 2019; Gaude and Frezza 2014). The molecular machinery that controls mitochondrial phenotype has been well described recently; however, the reciprocal relationship between mitochondrial morphology and metabolism is poorly understood (Pendin et al. 2017).

Our study aimed to investigate alterations in mitochondrial biogenesis and its relationship with mitochondrial morphology in pediatric acute lymphoblastic leukemia samples. TEM was used to examine the structural characteristics of mitochondria in leukemic blasts and healthy lymphocytes. In concordance with the EM results that we obtained, the mitochondria of lymphoblasts occupied a relatively larger area in the cytoplasm as compared to mitochondria from lymphocytes of healthy controls. The OXPHOS subunits form supercomplexes involved in mitochondrial respiration. Mitochondria organize their cristae dynamics at the ultrastructural level and a correlation has been observed between core cristae-shaping machinery, the emergence of cristae, and the OXPHOS system (Cogliati et al. 2016). Cogliati et al., have also highlighted the importance of ‘form and function’ concerning cristae shape and mitochondrial function. They have implied that respiratory efficiency is determined by mitochondrial cristae shape via its role in regulating the assembly of respiratory supercomplexes (Cogliati et al. 2013). Our data showed that the cristae of mitochondria in lymphoblasts of ALL patients were more compact and dense compared to that in mitochondria from healthy controls’ lymphocytes. Cristae cross-sectional area was also significantly higher in mitochondria of ALL patients as compared to controls. This could be attributed to the compaction of the inner mitochondrial membrane to form a condensed morphology to support the metabolic demands of the leukemic cell. It has been earlier reported that mitochondria of hypoxia-tolerant cells appear to have a condensed appearance which indicates their competence to produce an adequate amount of ATP by mitochondrial respiration (Arismendi-Morillo 2009).

A defective ATP synthase combined with an increase in mitochondrial biogenesis regulating factors (PGC-1α related co-activators i.e. PRC) has been evaluated in oncocytic models, which is also suggestive of the fact that decreased mitochondrial function is compensated by an increase in mitochondrial biogenesis to support the bioenergetic demands of the proliferating tumor (Gasparre et al. 2013). With the advent of TEM, papers have reported the morphology of leukemic cells and the variation in mitochondrial morphology (Sharp et al. 1979; Eguchi et al. 1987). However, since a role for mitochondrial dysfunction and mitochondrial dynamics in cancer is strongly postulated, it becomes equally important to investigate the changes taking place at the cristae level to get a proper insight into the causes and consequences of mitochondrial dysfunction. Although studies on other cancers report cristae changes (Lee et al. 2015; Signorile et al. 2019; Novotný et al. 2013), no previously published study has described these ultrastructural changes at mitochondrial cristae level in ALL and their possible link with mitochondrial biogenesis.

Mitochondria maintain homeostasis via a quality control mechanism involving a balance between mitochondrial biogenesis and mitophagy. A higher gene expression of *TFAM* and *NRF-1* has been reported in CLL patients relative to normal lymphocytes (Carew et al. 2004). Similar findings have been elucidated by Skrtic et al., demonstrating that acute myeloid leukemia (AML) cells have increased mitochondrial biogenesis and basal oxygen consumption compared to normal hematopoietic cells (Škrtić et al. 2011). We found a significantly higher gene expression of *TFAM* and *POLG* in lymphoblasts of ALL patients as compared to mononuclear cells in healthy controls. These differences in gene expression of *TFAM* and *POLG* could result from elevated *PGC-1α* gene expression since it is the master regulator of mitochondrial biogenesis. The median *PGC-1α* gene expression was also higher in lymphoblasts of ALL patients as compared to mononuclear cells in controls. The *PGC-1α* expression showed a positive correlation trend with both the M/C area and cristae cross-sectional area*; but* these trends were in opposite direction with c-myc expression. A study from our group has recently reported the correlation between *PGC-1α* gene expression and mtDNA copy number in AML patients; while *PGC-1α* inhibition was shown to decrease mtDNA copy number, inhibition of c-*myc* showed no significant effect (Chaudhary et al. 2021). We observed a strong positive correlation between the expressions of *PGC-1α* and *TFAM*. The concurrent correlations observed between *PGC-1α*and *TFAM* and mitochondrial morphological parameters support the premise of increased cristae remodelling and a role for these genes in cristae remodeling. The trends observed in the correlation of *PGC-1α* gene expression with the morphological parameters could become stronger if evaluated in a larger group of patients. *PGC-1α* gene expression has been linked to maintenance of bioenergetic function in in-vitro and clinical studies (Villena 2015; Kelly 2004). The expression of *PGC-1α* and *TFAM* has been shown to vary between tumors and even between tumor subtypes, and evolving evidence indicates a role in chemoresistance, metastasis, survival etc. (Gabrielson et al. 2014; Andrzejewski et al. 2017). The expression of *PGC-1α* could be important in the maintenance of tumor mitochondrial function especially when *myc* related pathways are dysregulated such as ALL. This is also borne out by the inverse relationship observed between these two genes with cristae parameters in our dataset and has been reported earlier with regard to gene expression (Yin et al. 2015; Sancho et al. 2015). It would be interesting to explore the dynamics of the relationship between *PGC-1α* and cristae morphology and examine if these linkages translate into prognostic implications for *PGC-1α* based on differential expression between samples, specifically in *myc* driven tumors like ALL.

Since we observed higher mtDNA copy numbers in patients, we also analyzed the expression of *CS* mRNA, a nuclear-encoded enzyme of the TCA cycle, as an indicator of functional mitochondria (Cai et al. 2017). Our data showed significantly higher *CS* gene expression in lymphoblasts in ALL patients as compared to mononuclear cells in controls which reflect the relative abundance of functional mitochondria. An increase in mitochondrial mass can be indicative of larger and/or more numerous mitochondria which have already been confirmed with transmission electron microscopy studies in AML (Sriskanthadevan et al. 2015). The *c-myc* gene expression was significantly higher in ALL patients as compared to controls which have an established role in regulating the mitochondrial structure and mitochondrial biogenesis (Li et al. 2005). A strong correlation was observed between *c-myc* and *CS* gene expression*.* These results collectively suggest increased biogenesis in lymphoblasts of ALL patients compared to lymphocytes of healthy controls. Similar to our findings, Sriskanthadevan et al., found increased mitochondrial biogenesis in AML assessed by gene expression and TEM findings (Sriskanthadevan et al. 2015).

These findings support the hypothesis that changes in cancer bioenergetics are expectedly accompanied by alterations in cristae morphology as a mechanism to maintain mitochondrial functionality. The observed changes could thus reflect adjustments to support the needs of the proliferating tumor. Ultrastructural images can offer definitive insight and a visual affirmation into the changes occurring at a subcellular organellar level. The image analysis and interpretation can be done using a combination of standard TEM instrumentation and freely available software. Thus, this technique, supported by routine molecular biology experiments can be used for exploring the modulation of the specific components of the cristae machinery and its link with oncogenic pathways, thus offering interesting insights into tumor maintenance mechanisms.

## Conclusions

Our study is the first to describe the mitochondrial morphological features at the cristae level in acute lymphoblastic leukemia. Increased mitochondrial biogenesis presented as an increase in the mitochondrial area along with changes at the ultrastructural level, suggesting a response of cristae to these biological stimuli. The data could be an entry point into the investigation of the significance of cristae morphology in mitochondrial function, and possible relationships with mitochondrial biogenesis in lymphoblastic leukemias. In summary, our results highlight alterations observed at the ultrastructural level in mitochondria in lymphoblasts of ALL patients which may be a response to the metabolic requirements of tumor cells.

*“Mitochondria came into the eukaryotic cell not only to exist but to ingeniously devise methods to direct cells towards their fate.”*

## Supplementary Information

**Additional file 1: Supplementary Table 1.** Primer sequences from minor arc region of mtDNA and beta-actin used for estimating mtDNA copy number in ALL patients and controls. **Supplementary Table 2.** The primer sequences for *TFAM, POLG, PGC-1α, c-myc, CS* and beta-actin for gene expression analysis in ALL patients and controls.

## Data Availability

The datasets used and/or analysed during the current study are available from the corresponding author on reasonable request.
