# Supplementary material for: Ultrastructural changes in cristae of lymphoblasts in acute lymphoblastic leukemia parallel alterations in biogenesis markers
Source: Appl Microsc. 2021 Dec 29;51:20. doi: 10.1186/s42649-021-00069-4 (PMC8716663; doi:10.1186/s42649-021-00069-4)
Supplement: Supplementary file 1 — Additional file 1: Supplementary Table 1. Primer sequences from minor arc region of mtDNA and beta-actin used for estimating mtDNA copy number in ALL patients and controls. Supplementary Table 2. The primer sequences for TFAM, POLG, PGC-1α, c-myc, CS and beta-actin for gene expression analysis in ALL patients and controls. [file 42649_2021_69_MOESM1_ESM.docx]

**Supplementary Materials**

**Supplementary Table 1:** Primer sequences from minor arc region of mtDNA and beta-actin used for estimating mtDNA copy number in ALL patients and controls.

**Supplementary Table 2:** The primer sequences for *TFAM, POLG, PGC-1α, c-myc, CS* and beta-actin for gene expression analysis in ALL patients and controls**.**

| Primer | Sequence (5’-3’) |
| --- | --- |
| \| Minor Arc Forward \| \| --- \| | \| CTAAATAGCCCACACGTTCCC \| \| --- \| |
| Minor Arc Reverse | AGAGCTCCCGTGAGTGGTTA |
| Beta-actin Forward (genomic DNA) | GATTCCTATGTGGGCGACGA |
| Beta-actin Reverse (genomic DNA) | CACCAGAAGAGGTAGCGGG |

**Table S1:** Primers for minor arc region and beta-actin in mtDNA for estimating mtDNA copy number in ALL patients and controls.

| **Primer** | **Sequence (5’-3’)** |
| --- | --- |
| \| Beta-actin Forward \| \| --- \| \|  \| | \|  \| CCTCGCCTTTGCCGATCC \| \| --- \| --- \| \|  \| |
| Beta-actin Reverse | CGCGGCGATATCATCATCC |
| *CS* Forward | AACTGCTACCCAAGGCTAAGG |
| *CS* Reverse | CTTTTGAGAGCCAAGATACCTGT |
| *TFAM* Forward | TCCCATAGTGCCTCGCTAGT |
| *TFAM* Reverse | CACAAAACTGAAGGGGGAGC |
| *POLG* Forward | CAGACCGGGAAGCACCGATT |
| *POLG* Reverse | CTCATGGTTGGTGCAGGGAC |
| *PGC-1α* Forward | CTTCTGGTACACAAGGCAATA |
| *PGC-1α* Reverse | TCCTTTCCTCGTGTCCACCA |
| *c-myc* Forward | TCAAGAGGCGAACACACAAC |
| *c-myc* Reverse | GGCCTTTTCATTGTTTTCCA |

**Table S2:** The primer sequences of *TFAM, POLG, PGC-1α, c-myc, CS* and beta-actin for mRNA gene expression in ALL patients and controls**.**
